# Nuclear mTORC1 Live-Cell Sensor nTORSEL Reports Differential Nuclear mTORC1 Activity in Cell Lines

**DOI:** 10.3390/ijms252212117

**Published:** 2024-11-12

**Authors:** Yifan Wang, Canrong Li, Yingyi Ouyang, Xiaoduo Xie

**Affiliations:** School of Medicine, Shenzhen Campus of Sun Yat-Sen University, Sun Yat-Sen University, Shenzhen 518107, China

**Keywords:** nuclear mTORC1, live-cell sensor, fluorescent reporter, PI3K-AKT-mTOR pathway, amino acid

## Abstract

The mammalian or mechanistic target of rapamycin complex 1 (mTORC1) is activated on the surface of lysosomes and phosphorylates substrates at various subcellular locations, including the lysosome, cytosol, and nucleus. However, the signaling and biological functions of nuclear mTORC1 (nmTORC1) are not well understood, primarily due to limited tools for monitoring mTORC1 activity in the nucleus. In this study, we developed a genetically encoded nmTORC1 sensor, termed nTORSEL, based on the phosphorylation of the eukaryotic initiation factor 4E (eIF4E) binding protein 1 (4EBP1) by mTORC1 within the nucleus. nTORSEL, like its predecessor TORSEL, exhibits a fluorescent punctate pattern in the nucleus through multivalent protein–protein interactions between oligomerized 4EBP1 and eIF4E when nmTORC1 activity is low. We validated nTORSEL using biochemical analyses and imaging techniques across representative cell lines with varying levels of nmTORC1 activity. Notably, nTORSEL specifically detects physiological, pharmacological, and genetic inhibition of nmTORC1 in mouse embryonic fibroblast (MEF) cells but not in HEK293T cells. Therefore, nTORSEL is an effective tool for investigating nuclear mTORC1 signaling in cell lines.

## 1. Introduction

The mammalian or mechanistic target of rapamycin (mTOR) is a highly conserved serine/threonine protein kinase controlling cell metabolism and growth [1,2]. Dysregulated mTOR activity is associated with cancer, diabetes, aging, and mTORopathies [3,4]. mTOR exists in two structurally and functionally distinct complexes: mTOR complex 1 (mTORC1) and mTOR complex 2 (mTORC2). The rapamycin-sensitive mTORC1 contains the catalytic mTOR kinase, regulatory-associated protein of mTOR (RAPTOR), mammalian lethal with SEC13 protein 8 (mLST8), DEP domain-containing mTOR-interacting protein (DEPTOR), and proline-rich Akt substrate of 40 kDa (PRAS40) [4,5]. As a central signaling hub, mTORC1 coordinates cellular anabolic and catabolic processes by phosphorylating key effectors, including eukaryotic initiation factor 4E (eIF4E) binding protein 1 (4EBP1), ribosomal S6 kinase (S6K), sterol regulatory element-binding protein 1 (SREBP1), carbamoyl-phosphate synthetase 2, aspartate transcarbamylase and dihydroorotase (CAD), and Unc-51-like kinase 1 (ULK1), etc. [6]. These effectors play critical roles in the synthesis of proteins, lipids, nucleotides, and autophagy induction [2,7].

mTOR is reported to be located in several distinct subcellular compartments, including the membranous surface of organelles, cytosol, and nucleus [8,9,10]. Mechanistic studies of lysosomal mTORC1 have laid the foundation for understanding mTORC1 signaling [2,11,12]. Under nutrient-rich conditions with amino acids (AAs) or glucose, the heterodimeric Rag GTPase complex RagA/B-RagC/D is tethered to the lysosomal surface by the LAMTOR/Ragulator complex, where it binds to Raptor and recruits mTORC1. The lysosomal mTORC1 is then activated by the allosteric binding of another lysosomal small GTPase RheB [13,14,15]. Rag and RheB GTPases are regulated by GTPase-activating proteins (GAPs), such as GATOR1 (GAP activity toward Rag 1) and TSC (tuberous sclerosis complex), which shuttle to and from the lysosomal surface in response to stimulating signals [14,16,17]. While GATOR1 inhibits RagA/B in response to AA or glucose sensing, the TSC complex inhibits RheB by integrating signals from the PI3K/AKT, LKB1/AMPK, Wnt/GSK3, and ERK/RSK pathways related to growth factor (GF), energy status, and stresses [3,18]. mTORC1 may also be regulated in other subcellular compartments besides the lysosome in a spatial- and temporal-specific manner [10,19].

Accumulating evidence suggests that mTOR functions in the nucleus, modulating RNA polymerase-directed gene expression related to cell growth and metabolism [8,9,20,21]. nmTORC1 signaling has been linked to cancer by modulating nuclear receptors such as AR and ERα [22,23,24]. Therefore, characterizing nmTORC1 signaling is pivotal for mechanistic studies and the development of novel therapies for mTOR-related diseases. Cytosolic mTORC1 signaling components such as AKT, RheB, Raptor, and S6K have also been found in the nucleoplasm of various cell lines [25,26,27,28]. However, it remains unclear whether the mTORC1 signaling components and regulatory mechanisms in the nucleus are similar to those in the cytosol or if nmTORC1 signaling is cell-type-specific. nmTORC1 activity was once thought to arise from translocated mTOR kinase and/or activated substrates from the cytosol [10,26]. Distinguishing locally activated nmTORC1 from translocated mTORC1 is challenging due to a lack of in situ detection tools. Methods for detecting nmTORC1 activity primarily rely on cell fractionation and immunochemical techniques using antibodies, which typically require disrupting or fixing cells under nonphysiological conditions. To overcome these limitations, fluorescence resonance energy transfer (FRET)-based mTORC1 live-cell reporters such as NLS-TORCAR, NLS-AIMTOR, and NLS-AKTSTOP have been developed. These reporters identified the nuclear pool of mTORC1 and revealed the non-canonical nmTOR functions [20,28,29,30]. However, minor changes in fluorescence ratios and the need for quantitative imaging analysis of single cells limit their application in high-throughput detection and tissue studies [29,30,31].

We recently developed an efficient cytosolic mTORC1 live-cell sensor known as TORSEL and successfully applied it in tissue and for high-throughput drug screening in living cells [32]. In this study, we upgraded TORSEL to a nuclear version called nTORSEL to visualize mTORC1 activity in the nucleus. This upgrade enables us to monitor differential nmTORC1 activity in various cell lines, expanding the toolkit for the spatiotemporal exploration of mTOR signaling.

## 2. Results

### 2.1. nTORSEL Senses nmTORC1 Activity in the Nucleus

**Figure 1:**
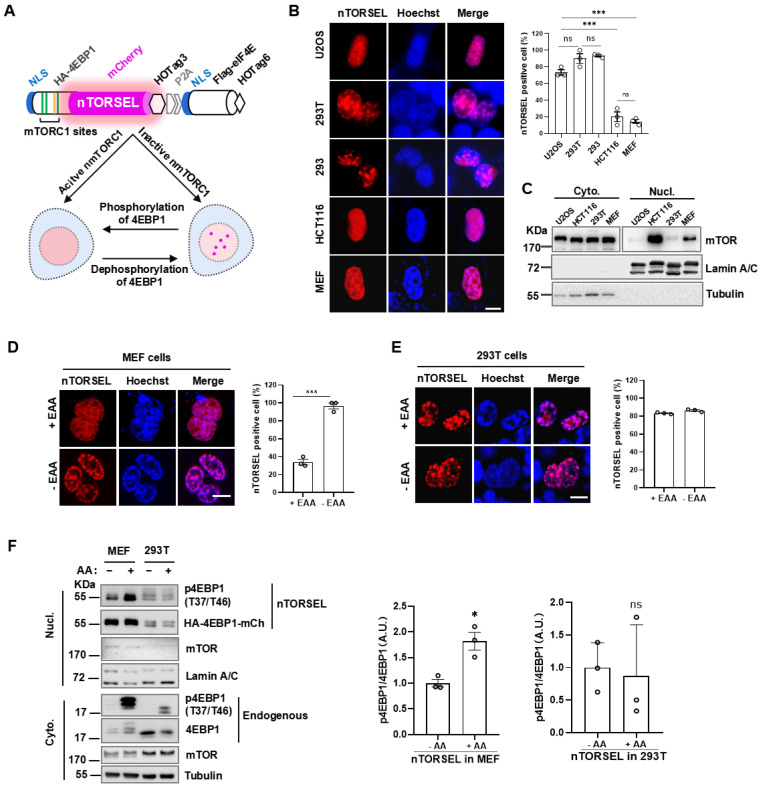
Design and characterization of nTORSEL. (**A**) Schematic diagram illustrating the structure of nTORSEL and its puncta formation in response to changes in mTORC1 activity within the nucleus. (**B**) Representative images of nTORSEL in U2OS, 293T, 293, HCT116, and MEF cells (left panel) and quantification of nTORSEL-positive cells (defined as > 5 puncta/cell) (right panel). (**C**) Immunoblot analysis of mTOR expression in the cytosolic (cyto.) and nuclear (nucl.) fractions for various cell lines. (**D**,**E**) Representative images and quantified nTORSEL signals in response to EAA starvation (12 h) in MEF and 293T cells. (**F**) Immunoblot analysis of 4EBP1(T37/T46) phosphorylation in nuclear and cytosolic fractions of nTORSEL-transfected MEF and 293T cells. Phosphorylation of nTORSEL was detected in the nuclear fraction, and endogenous 4EBP1 phosphorylation was detected in the cytosol (left panel). 4EBP1 phosphorylation was quantified by p4EBP1 (T37/T46)/4EBP1 signal ratios and normalized to the first control lane (=1.0). The quantified image data are presented as the mean ± SEM from three experiments, with approximately 50 cells analyzed for the percentage of nTORSEL-positive cells in each sample. Scale bar, 10 μm. Statistical analysis was performed using a two-tailed, unpaired Student’s *t*-test; ns, no statistical significance, * *p* < 0.05, and *** *p* < 0.001.

We previously devised a fluorescent biosensor called TORSEL (mTORC1 sensor for live cells), which changes its fluorescence pattern from diffuse to punctate through multivalent protein–protein interactions between oligomerized 4EBP1 and eIF4E when nmTORC1 activity is deficient [32]. TORSEL is primarily localized in the cytosol; however, mTORC1 components and phosphorylated substrates were also detected in the nucleus [6,10]. To visualize mTORC1 activity in the nucleus, we added the SV40 nuclear localization signal (NLS) to the N-terminal of each part of TORSEL, resulting in the nuclear expression of the reporter, called nTORSEL (nuclear TORSEL) (Figure 1A). Notably, nTORSEL exhibited a diffuse pattern in the nuclei of HCT116 and MEF cells, while it displayed a consistent punctate pattern in the nuclei of U2OS, HEK293 (293), and HEK293T (293T) cells (Figure 1B). nTORSEL showed a punctate pattern in both 293T and 293 cells, which are widely used in mTOR signaling studies (Figure 1B). This suggests that the SV40 large T antigen in 293T does not significantly impact mTORC1 signaling, as previously reported [33]. These results indicate that nuclear mTORC1 activity exists in HCT116 and MEF cells but is deficient in U2OS, 293, and 293T cells. Biochemical fractionation analysis revealed much higher levels of nuclear mTOR protein in MEF and HCT116 cells compared to U2OS and 293T cells (Figure 1C), as previously reported [26,27]. Consistently, nTORSEL responded to essential amino acid (EAA) starvation in MEF cells but not in 293T cells (Figure 1D,E). The results were validated by nucleus/cytoplasm fractionation analysis of MEF and 293T cells (Figure 1F), which showed that amino acid-induced mTORC1 phosphorylation of 4EBP1 in the nucleus occurred in MEF cells but not in 293T cells. Therefore, nTORSEL specifically senses and discriminates nuclear mTORC1 activity across various cell lines.

### 2.2. nTORSEL Specifically Responds to nmTORC1-Mediated 4EBP1 Phosphorylation

**Figure 2:**
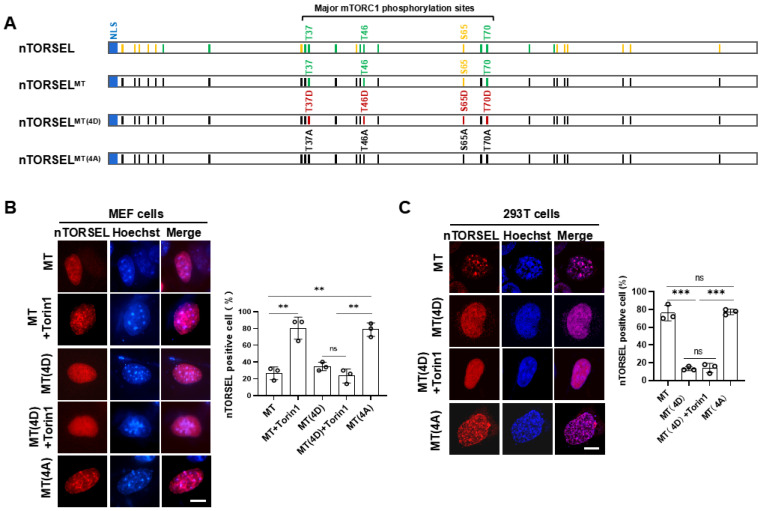
nTORSEL responds to mTORC1 phosphorylation of 4EBP1 in the nucleus. (**A**) Aligned sequences of 4EBP1 and its mutants in nTORSELs. Twenty-six Thr/Ser or mutated sites are marked as colored vertical lines within the box (Thr, green; Ser, yellow; Ala, black; and Asp, red). Four bracketed sites indicate major mTORC1 phosphorylation sites. (**B**) Representative images of nTORSEL^MT^ and related mutants in MEF cells treated with Torin1 (50 nM, 12 h) (left panel) and quantification of nTORSEL-positive cells (right panel). (**C**) Representative images of nTORSEL^MT^ and related mutants in 293T cells treated with Torin1 (left panel) and quantification of nTORSEL-positive cells (right panel). The quantified data are presented as the mean ± SEM from three experiments, with approximately 50 cells analyzed for the percentage of nTORSEL-positive cells in each sample. Scale bar, 10 μm. A two-tailed, unpaired Student’s *t*-test was used for statistical analysis; ns, no statistical significance, ** *p* < 0.01, and *** *p* < 0.001.

To confirm whether nTORSEL specifically senses nmTORC1 activity, we compared the 4EBP1 phospho-mutants derived from nTORSEL in MEF and 293T cells. Additional mTORC1-regulating sites, either directly or indirectly, besides the four major mTORC1 phosphorylation sites of 4EBP1, may affect the formation of TORSEL puncta [32]. Therefore, we chose nTORSEL^MT^ as an equivalent to nTORSEL to test the specificity of the reporters, as TORSEL^MT^ contains only the four major mTORC1 sites in 4EBP1 with all other Thr/Ser residues mutated to Ala (Figure 2A), and it has been shown to behave identically to TORSEL [32]. Further mutation of Thr37, Thr46, Ser65, and Thr70 into Asp or Ala created nTORSEL^MT(4D)^ or nTORSEL^MT(4A)^, representing the phosphomimetic or nonphosphorylatable forms of nTORSEL^MT^, respectively. As expected, nTORSEL^MT^ responded to mTOR kinase inhibitor Torin1, while nTORSEL^MT(4D)^ did not. Additionally, nTORSEL^MT(4A)^ constitutively formed puncta without mTOR inhibition (Figure 2B). These results suggest that nTORSEL responded to mTORC1 inhibition in MEF cells. In 293T cells, both nTORSEL^MT^ and nTORSEL^MT(4A)^ constitutively formed puncta with or without mTORC1 inhibition, while nTORSEL^MT(4D)^ did not form puncta even with Torin1 inhibition (Figure 2C). These results suggest that the constitutive puncta formation of nTORSEL^MT^ in the 293T nucleus was due to a lack of nuclear mTORC1 activity, and the formation of nTORSEL puncta is a 4EBP1 phosphorylation-specific event in response to mTORC1 inhibition in the nucleus.

### 2.3. nTORSEL Responds to the Inhibition of Growth Factor Signaling in MEF Cells

**Figure 3:**
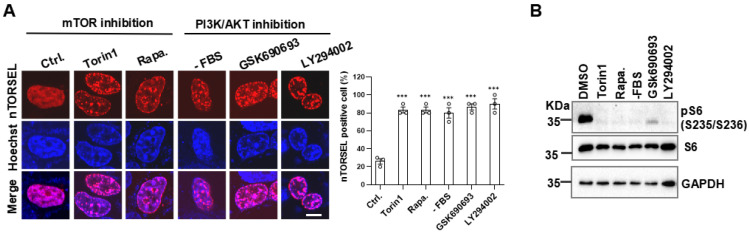
nTORSEL responds to inhibition of GF signaling in MEF cells. (**A**) Representative images of nTORSEL responding to PI3K/AKT/mTOR signaling inhibitors. nTORSEL-transfected MEF cells were treated as indicated (50 nM Torin1, 100 nM rapamycin, FBS starvation, 300 nM GSK690693, and 50 µM LY294002 for 12 h) (left panel) and quantified for nTORSEL-positive cells (right panel). (**B**) Immunoblot validation of mTORC1 inhibition by S6 phosphorylation in 293T cells treated as described in (**A**). The quantified data are presented as the mean ± SEM from three experiments, with approximately 50 cells analyzed for the percentage of nTORSEL-positive cells in each sample. Scale bar, 10 μm. Statistical analysis was performed using a two-tailed, unpaired Student’s *t*-test; *** *p* < 0.001.

To further assess the efficiency of nTORSEL in response to growth factor (GF) signaling, we tested its response to GF signaling inhibition using chemical inhibitors in MEF cells. nTORSEL effectively detected the suppression of mTORC1 caused by both serum starvation and PI3K-AKT inhibitor treatments that blocked GF signaling, as well as mTOR kinase inhibitor treatments (Figure 3A,B). When combined with the responses of nTORSEL to AA signaling (Figure 1D), these results indicate that nTORSEL is an effective tool for assessing nuclear mTORC1 inhibition through AA and GF signaling in MEF cells.

### 2.4. nTORSEL Reveals the Defective nmTORC1 Signaling Pathway in 293T Cells

**Figure 4:**
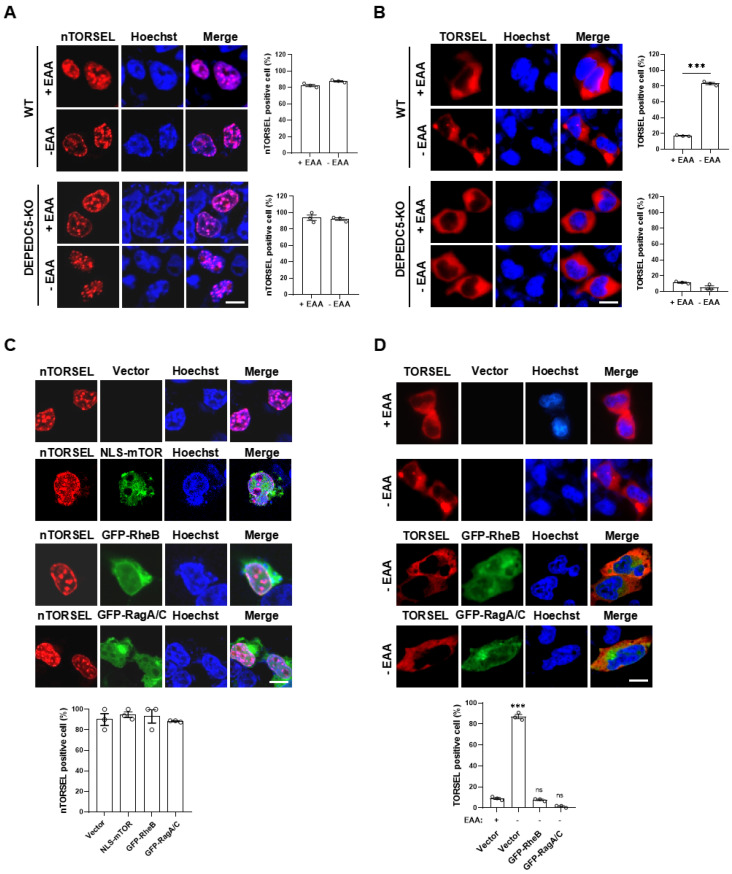
nTORSEL reveals defective nmTORC1 signaling in 293T cells. (**A**) Responses of nTORSEL to EAA starvation in 293T wild-type (WT) and DEPDC5-KO cells (left panel) and quantified nTORSEL-positive cells (right panel). (**B**) Responses of TORSEL to EAA starvation in 293T wild-type (WT) and DEPDC5-KO cells (left panel) and quantified TORSEL-positive cells (right panel). (**C**) Responses of nTORSEL to the overexpression of flag-tagged NLS-mTOR, GFP-RheB, and GFP-RagA + GFP-RagC in 293T cells (upper panel) and quantified nTORSEL-positive cells (lower panel). Flag-tagged NLS-mTOR was visualized by immunofluorescence staining with anti-flag antibody. (**D**) Responses of TORSEL to the overexpression of GFP-RheB and GFP-RagA + GFP-RagC in 293T cells (upper panel) and quantified TORSEL-positive cells (lower panel). The quantified data are presented as the mean ± SEM from three experiments, with approximately 50 cells analyzed for the percentage of nTORSEL-positive cells in each sample. Scale bar, 10 μm. A two-tailed unpaired Student’s *t*-test was used for statistical analysis; ns, no statistical significance, and *** *p* < 0.001.

In contrast to MEF, the 293T cell line represents nmTORC1-defective cells in this and previous reports [26,27]. To investigate the intrinsic factors involved in the inactivation of nmTORC1 in 293T cells, we first compared the cytosolic and nuclear mTORC1 activity using TORSEL and nTORSEL, respectively. TORSEL exhibited a diffuse signal pattern in the cytosol when cultured in EAA-containing medium, switching to a punctate pattern under EAA starvation. In contrast, nTORSEL remained in a punctate state regardless of EAA stimulation (Figure 4A,B upper panels). In DEPDC5-knockout cells with disrupted GATOR1 complex, TORSEL diffused and did not respond to EAA starvation, indicating AA-insensitive activation of cytosolic mTORC1. However, nTORSEL remained in a punctate state, suggesting a lack of nmTORC1 activity downstream of AA signaling (Figure 4A,B lower panels). Notably, the reconstitution of mTOR kinase by expressing an NLS-fused mTOR in the nucleus did not restore nuclear mTORC1 activity (Figure 4C). Additionally, overexpression of two key activating GTPases, RheB or RagA/RagC, both expressed in the cytosol and nucleus, did not disperse the nTORSEL puncta even in the presence of EAA; however, it did disperse the EAA starvation-induced cytosolic TORSEL puncta (Figure 4C,D). These results suggest that nmTORC1 signaling is defective and that activated cytosolic mTORC1 cannot shuttle into the nucleus in 293T cells.

## 3. Discussion

The compartmentalization of mTORC1 may be a fundamental principle for precise control of cell metabolism and growth, allowing for specific and efficient regulation of signal inputs. Nuclear mTORC1 signaling plays important roles in gene transcription, macromolecule synthesis, and mitochondrial regulation [21,34,35]. To investigate nuclear-specific mTORC1 activity in physiological and pathological contexts, we need effective tools to detect local nmTORC1 activity in living cells.

FRET- or BRET-based nmTORC1 sensors such as NLS-TORCAR or NLS-AIMTOR are valuable tools for monitoring compartmentalized nmTORC1 activity [29,30]. However, these reporters have limitations, including complexity in imaging and analysis, low signal-to-noise ratios, and issues with autofluorescence and light scattering in tissues. To address these challenges, we developed nTORSEL, an upgraded version of the cytosolic TORSEL. nTORSEL is exclusively expressed in the nucleus and specifically responds to nuclear mTORC1 inhibition in certain cell lines, such as MEF and HCT116 (Figure 1B). Notably, our results showed that nmTORC1 activity is not consistently detectable in all cell lines, as confirmed by distinct nTORSEL responses among them (Figure 1B–F). Specifically, we found that nTORSEL responds to nmTORC1-mediated 4EBP1 phosphorylation in MEF cells but not in 293T cells (Figure 2B,C). Inhibition of either GF signaling or AA signaling induced the transition of nTORSEL from a diffuse state to a punctate state in MEF cells, but not in 293T cells (Figure 1B–F and Figure 3). The relatively high background puncta of nTORSEL (approximately 20%) in MEF cells may be attributed to the low nmTORC1 activity compared to the TORSEL cytosolic background puncta, which is typically less than 10% [32] (Figure 3A). The deficiency of nmTORC1 activity does not appear to be due to a lack of mTOR kinase or its upstream GTPases RheB or RagA/C in the nucleus (Figure 4C,D). It may result from a deficiency of multiple components necessary for mTORC1 complex assembly or upstream signaling. Interestingly, cytosolic mTORC1 does not shuttle into the nucleus in 293T cells (Figure 4A,B), supporting the idea that mTORC1 is locally activated in the nuclei of MEF and other cell lines [20,28,29]. Nevertheless, further investigation is required to understand the differential nmTORC1 signaling among cell lines.

**Figure 5:**
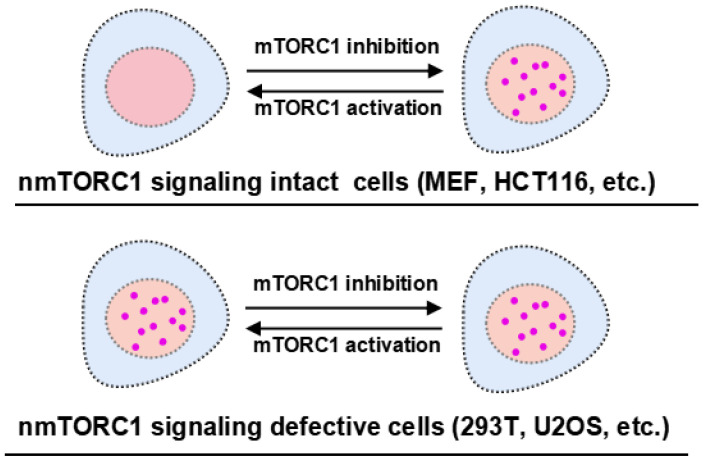
nTORSEL detects differential mTORC1 activity in the nucleus. The response model of nTORSEL is illustrated in cell lines grouped by the integrity of nmTORC1 signaling. In MEF or HCT116 cells, the nuclear mTORC1 signaling components are intact, allowing nTORSEL to switch from a diffuse state to a punctate pattern in response to mTORC1 inhibition. In 293T or U2OS cells, nmTORC1 activity is undetectable due to defective nmTORC1 signaling components, resulting in a constitutive punctate pattern for nTORSEL regardless of changes in cytosolic mTORC1 activity.

Collectively, nTORSEL has unique features that are beneficial for studying nmTORC1 signaling. It effectively detects changes in nmTORC1 activity in cell lines with functional nmTORC1 signaling, such as MEF cells. Additionally, it serves as a convenient tool for assessing the integrity of nmTORC1 signaling across different cell lines (Figure 5).

## 4. Materials and Methods

### 4.1. Cell Culture and Treatments

HEK293T (293T), HEK293 (293), U2OS, HCT116, and MEF cells were originally obtained from ATCC. The 293T DEPDC5-KO cell lines were generated as previously described [32]. All cells were cultured in DMEM supplemented with 10% FBS, 100 μg/mL streptomycin, and 100 U/mL penicillin at 37 °C with 5% CO_2_. Cell transfections were performed using polyethyleneimine (PEI) or Lipofectamine 3000 according to the manufacturer’s protocols. For serum starvation, cells were washed twice with PBS and then cultured for 12 h in DMEM without FBS. For EAA starvation, cells were cultured overnight in custom-ordered AA-free DMEM supplemented with NEAA, followed by the addition of EAA for stimulation. For whole-AA starvation, cells were cultured in AA-free DMEM for 50 min, and then 1xEAA/NEAA mixture was added for stimulation. All cell lines were confirmed to be free of mycoplasma. Detailed reagent information is listed in Supplementary Table S1.

### 4.2. Molecular Cloning and Plasmids

nTORSEL and related mutants were generated by PCR techniques through fusing SV40 NLS (KRPAATKKAGQAKKKK) to the N-terminal of HA-4EBP1 and Flag-eIF4E using TORSEL or TORSEL mutant plasmids as the templates [32]. The oligos used for NLS-fused mTOR subcloning PCR are listed in Supplementary Table S1. pEGFP-RheB, pEGFP-RagA, pEGFP-RagC, and pCAG-NLS-mTOR-Flag plasmids were kindly provided by Professor Kun-Liang Guan’s lab. All subcloning plasmids were confirmed by Sanger sequencing.

### 4.3. Antibodies and Chemical Reagents

Antibodies and reagents are listed in Supplementary Table S1.

### 4.4. Immunoblotting and Immunofluorescence Staining

IB and IF experiments were performed as previously described [32,36]. Briefly, cells were washed twice with ice-cold PBS and lysed with EBC buffer (50 mM Tris (pH 7.5), 120 mM NaCl, 0.5% NP-40) containing 1.0 mM dithiothreitol (DTT), protease inhibitors, and phosphatase inhibitors. Protein concentrations were measured using Bradford or BCA reagents (Beyotime Biotechnology, Shanghai, China), resolved by 10% or 12% SDS–PAGE, and analyzed by immunoblotting with the indicated antibodies. For anti-flag IF, transfected cells were seeded on polylysine-coated glass coverslips and grown overnight, then fixed with 4% paraformaldehyde (PFA) in PBS for 10 min and permeabilized with 0.1% Triton X-100 in PBS for 5 min. After blocking with 5% bovine serum albumin (BSA), cells were incubated with anti-Flag antibodies and subsequently with secondary antibodies conjugated to Alexa Fluor 488.

### 4.5. Cytosolic and Nuclear Protein Fractionation

Cell fractionation was performed as previously described [26]. Briefly, cell pellets were lysed in buffer A (20 mM Tris-HCl pH 7.6, 0.6% CHAPS, 0.1 mM EDTA, and 2.0 mM MgCl_2_ supplemented with protease and phosphatase inhibitors) for 2 min at room temperature and then another 10 min on ice. Samples were homogenized by passing through a 20-gauge needle five times. Nuclei were pelleted by centrifugation at 600× *g* for 5 min at 4 °C, and the supernatant containing the cytoplasmic proteins (cyto.) was collected and stored at −80 °C. The remaining nuclei were washed three times with buffer A and lysed in buffer B (20 mM HEPES pH 7.9, 0.4 M NaCl, 2.5% glycerol, and 1.0 mM EDTA supplemented with protease and phosphatase inhibitors) on ice for 30 min, followed by sonication (3 × 10 s each at 20% output). Supernatants containing soluble nuclear proteins (nucl.) were collected by centrifugation at 20,000× *g* for 20 min and stored at −80 °C.

### 4.6. Live-Cell Microscopic Imaging and Quantification

The cells were grown in 4-chamber glass-bottom microwell dishes (Cellvis, D35C4-20-1-N). After transfection with TORSEL or other indicated plasmids, live-cell imaging was performed using a Nikon Eclipse Ti2 inverted microscope with a 60x oil objective. For quantifying nTORSEL- or TORSEL-positive cells, images were acquired from three experiments. Approximately 50 cells from each sample were analyzed for the percentage of nTORSEL- or TORSEL-positive cells, defined as those containing more than five visible puncta in the nucleus.

### 4.7. Quantification and Statistical Analysis

ImageJ and GraphPad Prism 9.0 were used to quantify the data as well as to create most of the graphics. Data from biological or technical replicates are presented with the standard error of the mean (SEM). Statistical analysis was performed using a two-tailed Student’s *t*-test, with * *p* < 0.05 considered statistically significant.

## 5. Conclusions

We have developed a unique genetically encoded fluorescent sensor to monitor nuclear mTORC1 activity in living cells. This reporter reveals that nuclear-compartmentalized mTORC1 signaling exists in some but not all cell lines, suggesting differential nuclear mTORC1 functions in the human body.

## Data Availability

The uncropped immunoblotting images are provided in Supplementary Figure S1. Other data that support the findings of this study are available from the corresponding author upon reasonable request.

## Supplementary Materials

The following supporting information can be downloaded at: https://www.mdpi.com/article/10.3390/ijms252212117/s1.
