# Supplementary material for: Nuclear mTORC1 Live-Cell Sensor nTORSEL Reports Differential Nuclear mTORC1 Activity in Cell Lines
Source: Int J Mol Sci. 2024 Nov 12;25(22):12117. doi: 10.3390/ijms252212117 (PMC11594266; doi:10.3390/ijms252212117)
Supplement: Supplementary file 1 [file ijms-25-12117-s001.zip › Supplementary figure S1.pdf]

Figure 1C

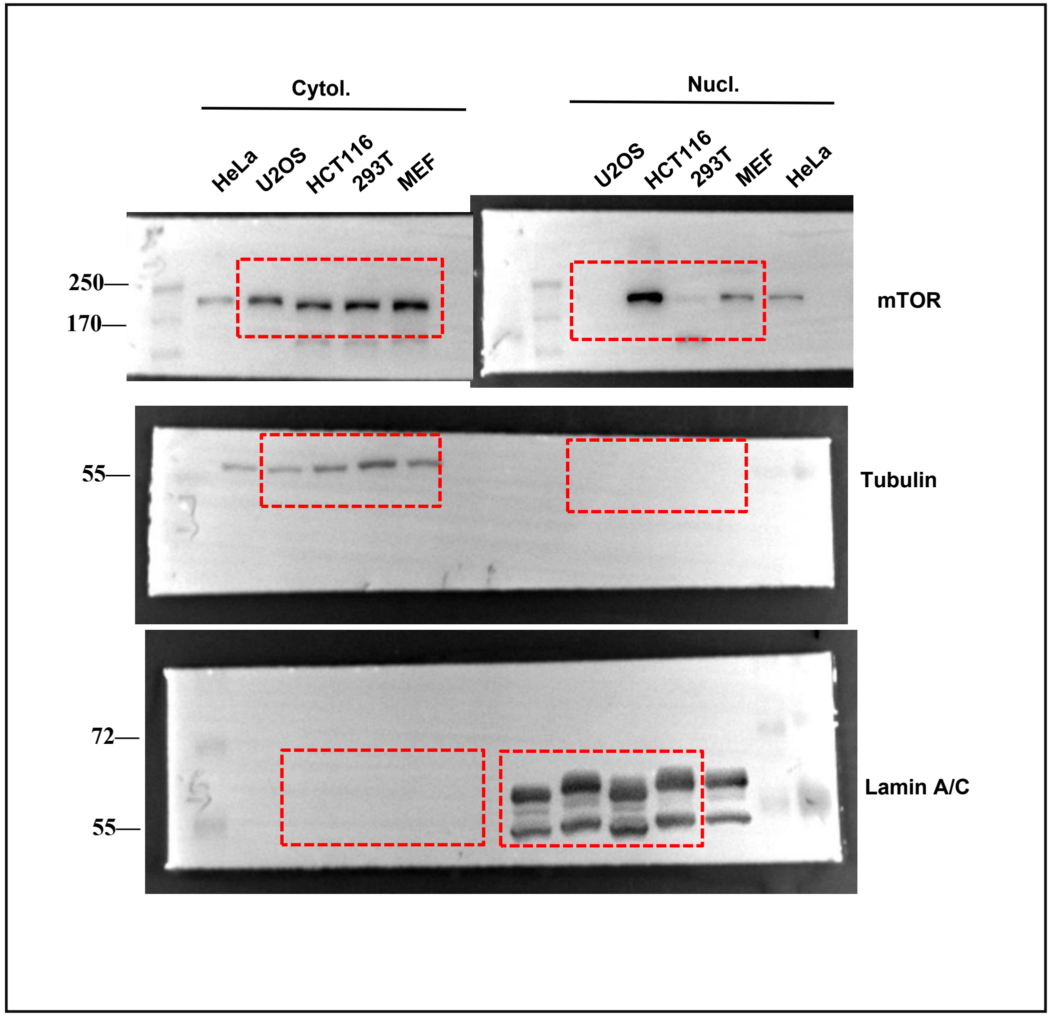

Figure 1F

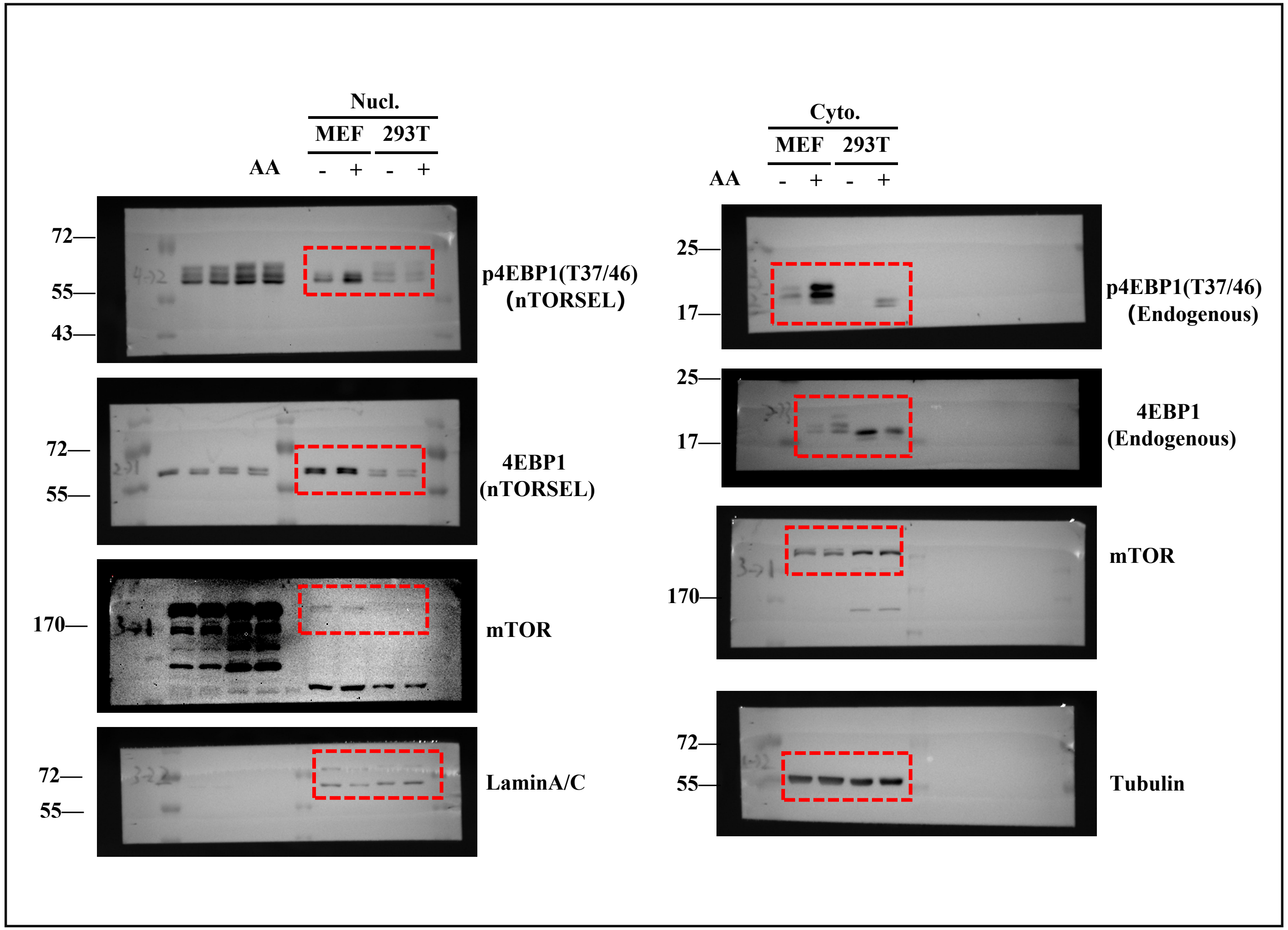

Figure 3B

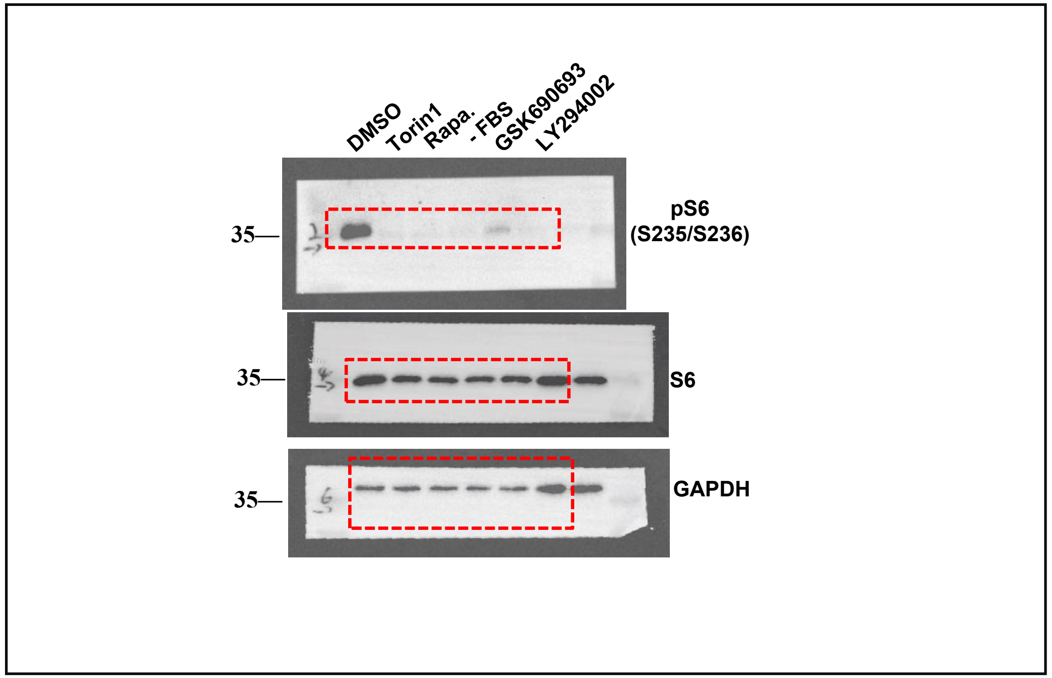
DMSO
Torin1
Rapa.
-FBS
GSK690693
LY294002
35—
pS6
(S235/S236)
35—
S6
35—
GAPDH
