# Supplementary material for: Nuclear mTORC1 Live-Cell Sensor nTORSEL Reports Differential Nuclear mTORC1 Activity in Cell Lines
Source: Int J Mol Sci. 2024 Nov 12;25(22):12117. doi: 10.3390/ijms252212117 (PMC11594266; doi:10.3390/ijms252212117)
Supplement: Supplementary file 1 [file ijms-25-12117-s001.zip › Supplementary Table S1.doc]

**Table S1: Information on antibodies, reagents and oligos.**

| **Reagent name** | **Source** | **Catalog number** |
| --- | --- | --- |
| **Primary antibodies** | | |
| anti-pS6(S235/S236) | Cell Signaling Technology | 4858S |
| anti-S6 | BETHYL | A300-557A |
| anti-4EBP1 | Cell Signaling Technology | 9644 |
| anti-p4EBP1(T37/46) | Cell Signaling Technology | 2855 |
| anti-HA | Cell Signaling Technology | 3724 |
| anti-Flag | Sigma-Aldrich | F7425 |
| anti-mTOR | Cell Signaling Technology | 2983T |
| anti-GAPDH | Sangon Biotech | AB10016 |
| anti-Tubulin | Invitrogen | 236-10501 |
| anti-LaminA/C | Cell Signaling Technology | 4777 |
| **Secondary antibody** | | |
| HRP-labeled Goat Anti-Mouse IgG(H+L） | Beyotime | A0216 |
| HRP-labeled Goat Anti-Rabbit IgG(H+L) | Beyotime | A0208 |
| Goat Anti-Rabbit IgG H&L (Alexa Fluor® 488) | abcam | ab150077 |
| **Compound and inhibitors** | | |
| Torin1 | Selleck | S2827 |
| Rapamycin | Selleck | S1039 |
| GSK690693 | Selleck | T6285 |
| LY294002 | TargetMol | T2008 |
| COmplete™ Protease Inhibitor | Roche | 4693132001 |
| Phosphatase inhibitor cocktail A | Beyotime | P1081 |
| **Cell culture and transfection reagents** | | |
| DMEM | Gibco | C11995500BT |
| AA free DMEM | BasalMedia | X014C |
| FBS | ExCell | FSP500 |
| MEM Amino Acids (100 X EAA) | Gibco | 11130077 |
| MEM Non-Essential Amino Acids (100X NEAA) | Gibco | 11140050 |
| Streptomycin and penicillin | Gibco | 15140122 |
| Lipofectamine 3000 | Invitrogen | L3000008 |
| polyethylenimine (PEI) | Sigma | 408727 |
| **Oligos sequences for NLS-mTOR PCR** | | |
| NLS-HA-F1 | 5’-ggttaagcttaagcgacctgccgccacaaagaaggctggacaggcta-3’ | |
| NLS-HA-F2 | 5’-aaagaaggctggacaggctaagaagaagaaagactacaaggacgatgacga-3’ | |
| 4EBP1-R | 5’-ggttaccggtgtgcctccaccgctgccagatccgccaatgtccatctcaaactgtg-3’ | |
| NLS-Flag-F1 | 5’-ggttaagcttaagcgacctgccgccacaaagaaggctggacaggcta-3’ | |
| NLS-Flag-F2 | 5’-aaagaaggctggacaggctaagaagaagaaagactacaaggacgatgacga-3’ | |
| eIF4E-R | 5’-ggttgtcgacaacaacaaacctatttttag-3’ | |
